# GM1 Ganglioside Promotes Osteogenic Differentiation of Human Tendon Stem Cells

**DOI:** 10.1155/2018/4706943

**Published:** 2018-08-23

**Authors:** Sonia Bergante, Pasquale Creo, Marco Piccoli, Andrea Ghiroldi, Alessandra Menon, Federica Cirillo, Paola Rota, Michelle M. Monasky, Giuseppe Ciconte, Carlo Pappone, Pietro Randelli, Luigi Anastasia

**Affiliations:** ^1^Laboratory of Stem Cells for Tissue Engineering, Scientific Institute for Research, Hospitalization, and Health Care (IRCCS) Policlinico San Donato, San Donato 20097, Italy; ^2^Arrhythmology Department, Scientific Institute for Research, Hospitalization, and Health Care (IRCCS) Policlinico San Donato, San Donato Milanese, Italy; ^3^Azienda Socio Sanitaria Territoriale Centro Specialistico Ortopedico Traumatologico Gaetano Pini-CTO, Milano 20122, Italy; ^4^Department of Biomedical Sciences for Health (L.I.T.A.), Università degli Studi di Milano, Segrate 20090, Italy

## Abstract

Gangliosides, the sialic acid-conjugated glycosphingolipids present in the lipid rafts, have been recognized as important regulators of cell proliferation, migration, and apoptosis. Due to their peculiar localization in the cell membrane, they modulate the activity of several key cell receptors, and increasing evidence supports their involvement also in stem cell differentiation. In this context, herein we report the role played by the ganglioside GM1 in the osteogenic differentiation of human tendon stem cells (hTSCs). In particular, we found an increase of GM1 levels during osteogenesis that is instrumental for driving the process. In fact, supplementation of the ganglioside in the medium significantly increased the osteogenic differentiation capability of hTSCs. Mechanistically, we found that GM1 supplementation caused a reduction in the phosphorylation of the platelet-derived growth factor receptor-*β* (PDGFR-*β*), which is a known inhibitor of osteogenic commitment. These results were further corroborated by the observation that GM1 supplementation was able to revert the inhibitory effects on osteogenesis when the process was inhibited with exogenous PDGF.

## 1. Introduction

Injuries to the tendon-to-bone enthesis are common in the field of orthopedic medicine, and high failure rates are often associated with their repair [1]. The use of biologic adjuvants that promote tissue regeneration, such as growth factors, platelet-rich plasma, and stem cells, have shown great potential for improving healing rates and function after surgery [2]. Accordingly, the use of tendon stem cells to improve tendon-bone junction repair has been considered advantageous, as tendon stem cells already belong to the tendon environment and possess the plasticity to potentially recover the different tissues found in the tendon-to-bone enthesis [3]. Along these lines, we reported the first isolation of human tendon stem cells from the supraspinatus and long head of the biceps tendons, and we demonstrated that they can be induced to differentiate toward osteoblasts, adipocytes, and muscle cells [4]. Nonetheless, an open issue in the stem cell field is to perfect the differentiation strategies in order to drive the process toward a specific phenotype and to avoid undesired cell commitment or, even more detrimental, the uncontrolled proliferation of undifferentiated progenitor cells. In this context, herein we investigated the role of gangliosides, which are sialic acid-containing glycosphingolipids (GSLs) ubiquitously distributed in cell membranes [5], in the osteogenic differentiation of hTSCs. Numerous studies have confirmed that gangliosides and their expression levels are controlled during development [6] and are cell type-specific [7], supporting the idea that these molecules are key players in cell commitment. While some biological roles of these lipids have been clearly recognized, as they have been shown to be involved in processes like cell proliferation [8], cell adhesion [9], apoptosis [10], and differentiation [11], less is known about their role in stem cell homeostasis and differentiation. Nonetheless, it has been shown that a reduction of ganglioside biosynthesis inhibits the neuronal differentiation of MSCs in the early stage of the process [12], and our group recently demonstrated that an increase of ganglioside GD1a is crucial for human bone marrow mesenchymal stem cell (MSC) differentiation [13]. Moreover, we demonstrated the pivotal role of sialidase NEU3 in regulating ganglioside GM3 content, which is a key in skeletal muscle cell differentiation and survival under hypoxia [14–17]. Clearly, as gangliosides are mainly distributed in the lipid rafts of cell plasma membranes, which are rich in key tyrosine kinase receptors, the present study further corroborates the notion that we are at the beginning of fully unveiling the role of these sphingolipids in stem cell biology.

## 2. Materials and Methods

### 2.1. Cell Isolation and Culture

Human tendon stem cells (hTSCs) were isolated from supraspinatus tendon specimens collected during arthroscopic rotator cuff repair, as previously reported [4]. The isolated hTSCs were cultured in minimal essential medium alpha modification (*α*-MEM) (Merck) supplemented with 2 mM L-glutamine (Euroclone), 1% antibiotic-antimycotic mixture (Euroclone), and 20% (*v*/*v*) fetal bovine serum (FBS) (HyClone, Thermo Fisher Scientific) at 37°C in a 5% CO_2_ and 95% air-humidified atmosphere. The medium was changed every 2-3 days.

### 2.2. Osteogenic and Adipogenic Differentiation

hTSCs were seeded at a concentration of 3 × 10^4^ cells/cm^2^ in a growth medium, and after 24 hours, cells were switched to an osteogenic or adipogenic medium for 17 days or 21 days, respectively. Osteogenic differentiation was obtained by culturing cells in the presence of DMEM-low glucose (Merck) supplemented with 4 mM L-glutamine (Euroclone), 1% antibiotic-antimycotic mixture (Euroclone), 10% FBS (HyClone, Thermo Fisher Scientific), 10 nM cholecalciferol (Merck Millipore), and the mesenchymal stem cell osteogenesis kit (Merck Millipore) according to the manufacturer's instructions. Adipogenic differentiation was induced by culturing cells in the presence of DMEM-low glucose supplemented with 4 mM L-glutamine, 1% antibiotic-antimycotic mixture, 10% FBS, and the mesenchymal stem cell adipogenesis kit (Merck Millipore), according to the manufacturer's instructions. To evaluate the effects of ganglioside GM1 treatment (Santa Cruz Biotechnology) on differentiation, hTSCs were cultured for 17 days in an osteogenic medium or 21 days in adipogenic medium supplemented with 1, 10, 50, and 100 *μ*M GM1. To evaluate the effects of the platelet-derived growth factor-BB (PDGF-BB, Thermo Fisher Scientific) on osteogenic differentiation, cells were cultured in an osteogenic medium containing PDGF-BB at the final concentration of 10 ng/ml. The differentiation medium was changed every 2-3 days.

### 2.3. Metabolic Radiolabeling of Cell Sphingolipids

The metabolic radiolabeling of cell sphingolipids was performed as previously described by Riboni et al. [18]. Briefly, [3-^3^H]-sphingosine (D-erythro > 97%, 50 *μ*Ci, 1.85 MBq, PerkinElmer) was dissolved in DMEM-low glucose with 10% FBS to a final concentration of 2.4 nM sphingosine, corresponding to 110.000 dpm/ml radioactivity. The medium was added to the cells and incubated for 2 hours (pulse) at 37°C, then it was replaced with DMEM-low glucose with 10% FBS without [^3^H]-sphingosine for 48 hours (chase). After the incubation, cells were harvested by cell scraping in phosphate-buffered saline (PBS). Cell suspensions were frozen and lyophilized.

### 2.4. Extraction and Chromatographic Separation of Radiolabeled Sphingolipids

Total lipid extraction was performed as previously described by Bergante et al. [13]. Briefly, lipids were first extracted with 20 : 10 : 1 (*v*/*v*) chloroform/methanol/water, dried under a nitrogen stream, and then a two-phase partitioning was carried out in chloroform/methanol 2 : 1 (*v*/*v*) and 20% (*v*/*v*) water. After partitioning, gangliosides of the aqueous phase were separated and analyzed by high-performance thin-layer chromatography (HPTLC), using as running solvent chloroform/methanol/0.2% aqueous CaCl_2_ 60 : 40 : 9 (*v*/*v*/*v*) [19, 20]. Radiolabeled sphingolipids were visualized with a Beta-Imager 2000 (Biospace). The radioactivity associated with individual lipids was determined with *β*-Vision software (Biospace).

### 2.5. RNA Extraction and Real-Time PCR

Total RNA was isolated using TRIzol Reagent (Ambion, Life Technologies), and 1 *μ*g of extracted RNA was reverse transcribed to cDNA using the iScript cDNA synthesis kit (Bio-Rad) according to the manufacturer's instructions. Real-time PCR was performed in a 96-well plate with 10 ng of cDNA as a template, 0.2 *μ*M primers, and 2x Power SYBR Green PCR Master Mix (Promega) in 20 *μ*L final volume per well, using a StepOnePlus Real-Time PCR System (Applied Biosystems). The following primers were used to amplify the corresponding target genes: human alkaline phosphatase (ALP) forward 5′-CGCACGGAACTCCTGACC-3′ and reverse 5′-GCCACCACCACCATCTCG-3′, peroxisome proliferator-activated receptor-*γ* (PPAR-*γ*) forward 5′-TTCCTTCACTGATACACTGTCTGC-3′ and reverse 5′-GGAGTGGGAGTGGTCTTCCATTAC-3′, lipoprotein lipase (LPL) forward 5′-AGAGAGAACCAGACTCCAATG-3′ and reverse 5′-GGCTCCAAGGCTGTATCC-3′, beta 1,3-galactosyltransferase (GM1 synthase) forward 5′-CGCCTTCCAGGACTCCTACC-3′ and reverse 5′-CCGTCTTGAGGACGTATCGG-3′, osteocalcin forward 5′-GCAGCGAGGTAGTGAAGAG-3′ and reverse 5′-GAAAGCCGATGTGGTCAGC-3′, and S14 (used as endogenous control in all real-time PCR experiments) forward 5′-GTGTGACTGGTGGGATGAAGG-3′ and reverse 5′-TTGATGTGTAGGGCGGTGATAC-3′.

### 2.6. Analysis of Mineralization

Matrix mineralization of hTSCs was evaluated at the 17th day of osteogenic differentiation using the osteogenesis assay kit (Merck Millipore). Briefly, cells were fixed with 4% paraformaldehyde at room temperature for 15 minutes. In order to detect mineral deposition in the extracellular matrix, cells were washed twice with PBS and incubated with alizarin red stain solution for 20 minutes. The dye was then extracted from the stained monolayer according to the manufacturer's instructions and quantified using a Victor 3 instrument (Perkin Elmer).

### 2.7. Immunoblotting

Cells were harvested in ice-cold PBS by cell scraping and centrifuged at 400 ×g for 10 minutes at 4°C. Cells were lysed in RIPA buffer (150 mM sodium chloride, 1% Triton X-100, 0.5% sodium deoxycholate, 0.1% sodium dodecyl sulphate, and 50 mM Tris pH 8) containing complete protease and phosphatase inhibitors (Merck). After cell lysis, the samples were centrifuged at 10,000 ×g for 15 minutes at 4**°**C. Protein amounts were measured using a Pierce BCA protein assay kit (Thermo Scientific). Proteins were loaded into a 10% SDS-PAGE gel, then transferred onto a nitrocellulose membrane (Trans-Blot, Bio-Rad Laboratories) by electroblotting. After blocking the membranes with 5% (*w*/*v*) of nonfat dry milk in Tris-buffered saline-Tween 0.1% (TBS-T) for 1 hour at room temperature, they were incubated overnight at 4°C with the following primary antibodies: rabbit phospho-PDGFR-*β*, 1 : 1000 dilution (Y751, Cell Signaling); rabbit PDGFR-*β*, 1 : 1000 dilution (Cell Signaling); and rabbit monoclonal early endosome antigen 1 (EEA1), 1 : 1000 dilution (Cell Signaling). The membranes were then washed in TBS-T three times and incubated for 1 hour at room temperature with specific secondary antibodies. In particular, phospho-PDGFR-*β* was incubated with the IRDye® 800CW goat anti-mouse IgG (LI-COR), the total PDGFR-*β* with the IRDye 680RD goat anti-rabbit IgG (Li-COR), and EEA1 with the HRP-conjugated anti-rabbit IgG (Amersham), diluted 1 : 5000 in 5% (*w*/*v*) nonfat dry milk in TBS-T. The membranes were analyzed by the Odyssey® FC imaging system (LI-COR), and the densitometric analysis was performed with the specific Image Studio™ software (LI-COR).

## 3. Results

### 3.1. Ganglioside Changes in hTSC Differentiation toward Osteoblasts and Adipocytes

**Figure 1:**
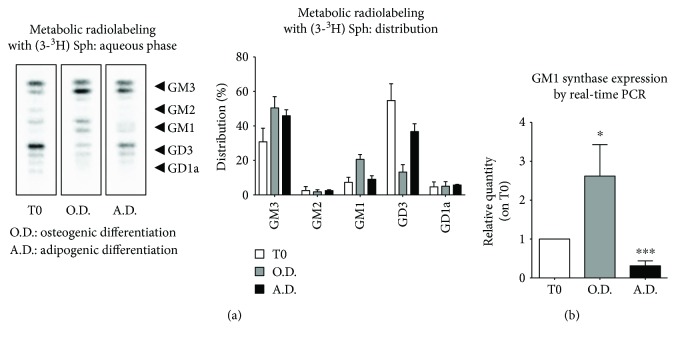
Ganglioside pattern upon differentiation of hTSCs to either osteoblasts or adipocytes. (a) Metabolic radiolabeled gangliosides separated by HPTLC and visualized with a Beta-Imager 2000 (Biospace). Doubled spots in cellular gangliosides correspond to the presence of species with different chain lengths of fatty acids. The graph on the right represents the percentage distribution of radiolabeled gangliosides. (b) Real-time PCR analysis of GM1 synthase gene expression in hTSCs differentiated toward osteoblasts (O.D.) or adipocytes (A.D.) as compared to that in undifferentiated cells (T0). Ribosomal protein S14 gene was used as housekeeper gene. All data are means ± SD of three different experiments. The statistical analysis was determined by Student's t-test. ^∗^*p* < 0.05, ^∗∗∗^*p* < 0.001.

To assess the ganglioside pattern distribution of hTSCs, cells were metabolically radiolabeled with the sphingolipid precursor [3-^3^H]-sphingosine and quantitatively analyzed by HTPLC coupled with a radiochromatoscanner, as described in “Materials and Methods.” The ganglioside distribution in proliferating hTSCs was as follows: GM3 (30.79% ± 7.85), GM2 (2.53% ± 2.33), GM1 (7.28% ± 2.94), GD3 (43.83% ± 19.35), and GD1a (4.71% ± 2.80), with GM3 and GD3 being the main gangliosides (Figure 1(a) and 1(b), T0).

Next, changes in ganglioside pattern were evaluated upon differentiation of hTSCs to either osteoblasts or adipocytes, as previously reported [4], by metabolic radiolabeling after 17 and 21 days of cell culturing in either osteogenic (O.D.) or adipogenic (A.D.) medium (Figure 1(a)). When hTSCs were differentiated toward osteoblasts, a 1.6- and 2.8-fold increase of GM3 and GM1 gangliosides was observed, respectively, as well as a 3.7-fold decrease of GD3, as compared to proliferating undifferentiated cells. When hTSCs were differentiated toward adipocytes, a 1.7-fold increase in GM3 and 1.5-fold decrease in GD3 relative distribution were observed, as compared to undifferentiated cells, while no significant changes in the relative quantity of GM1 could be observed (Figure 1(a)). To test whether the observed increase of GM1 during osteogenesis was due to an upregulation of its biosynthesis, GM1 synthase expression was measured by real-time PCR, and a 2.6-fold increase could be observed at the end of the differentiation process, as compared to proliferating hTSCs. On the other hand, a 3.2-fold reduction of GM1 synthase expression was measured when hTSCs were induced to differentiate toward adipocytes (Figure 1(b)).

### 3.2. Effects of Exogenous GM1 on Osteogenic Differentiation of hTSCs

**Figure 2:**
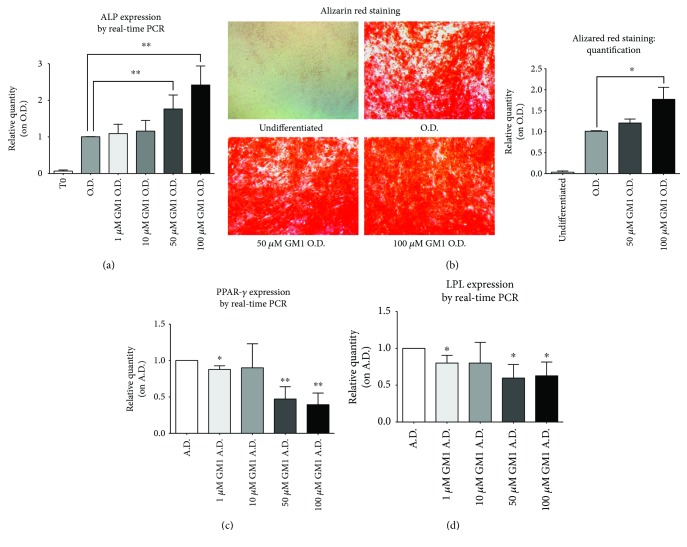
Evaluation of hTSC differentiation either to osteoblasts and adipocytes upon GM1 treatment. (a) Gene expression of the osteogenic marker ALP by real-time PCR. hTSCs were differentiated toward osteoblasts for 17 days in osteogenic medium supplemented with exogenous 1, 10, 50, and 100 *μ*M GM1. The results were compared to hTSCs differentiated in GM1-free osteogenic medium (O.D.). Ribosomal protein S14 gene was used as endogenous control. (b) Analysis and quantification of calcium deposits in hTSCs after osteogenic differentiation by alizarin red staining. Undifferentiated hTSCs and hTSCs differentiated in the presence of 50 *μ*M and 100 *μ*M GM1 were compared to hTSCs differentiated in GM1-free osteogenic medium (O.D.) and considered as controls. (c, d) Gene expression analysis of adipogenic markers, PPAR-*γ* and LPL, by real-time PCR. hTSCs were differentiated toward adipocytes for 21 days in adipogenic medium supplemented with exogenous 1, 10, 50, and 100 *μ*M GM1. The results were compared to hTSCs differentiated in GM1-free adipogenic medium (A.D.). Ribosomal protein S14 gene was used as endogenous control. All data are means ± SD of four different experiments. The statistical analysis was determined by Student's t-test. ^∗^*p* < 0.05, ^∗∗^*p* < 0.01.

To test the role of GM1 increase during osteogenesis, exogenous 1, 10, 50, and 100 *μ*M GM1 was supplemented in the osteogenic medium during the differentiation process. Osteogenic marker ALP gene expression was measured by real-time PCR after 17 days of differentiation and compared to undifferentiated cells (T0) and GM1-free osteogenic medium (O.D.). Results showed a significant 1.8- and 2.4-fold increase in ALP expression when cells were supplemented with 50 or 100 *μ*M GM1 in addition to the osteogenic medium, respectively, as compared to O.D. (Figure 2(a)).

Afterward, cells were induced to differentiate to osteoblasts in the presence of 50 or 100 *μ*M GM1 and were evaluated for their capacity to sustain the mineralization of the extracellular matrix using a standard alizarin red staining, as described in “Materials and Methods.” Dye relative quantification showed an increase of red staining in hTSCs differentiated in the presence of GM1, which was significantly higher (1.7-fold) in 100 *μ*M GM1-treated cells (Figure 2(b)). On the contrary, exogenous GM1 strongly inhibited the gene expression of the adipogenic markers LPL and PPAR-*γ* (Figures 2(c) and 2(d)).

### 3.3. Mechanism of GM1-Activated Osteogenesis

**Figure 3:**
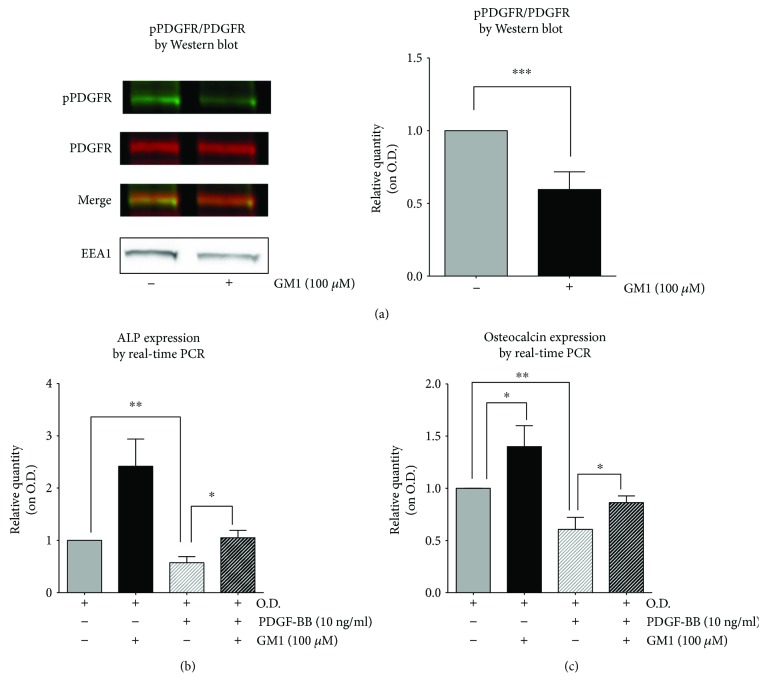
Effects of GM1 treatment on PDGFR activation. (a) Western blot analysis and quantification of PDGFR-*β* activation. hTSCs were differentiated toward osteoblasts in osteogenic medium supplemented with 100 *μ*M GM1, as compared to hTSCs differentiated in GM1-free osteogenic medium (O.D.). Total proteins were extracted and analyzed with anti-phosphorylated-PDGFR-*β* (Tyr 751) antibody (green) and anti-PDGFR-*β* (28E1) antibody (red). EEA1 expression was used as internal control. Data are means ± SD of four different experiments. (b, c) Gene expression analysis of the osteogenic markers ALP and osteocalcin by real-time PCR. hTSCs were differentiated toward osteoblasts in osteogenic medium supplemented with 100 *μ*M GM1 or 10 ng/ml PDGF-BB or with both 100 *μ*M GM1 and 10 ng/ml PDGF-BB. The results were compared to hTSCs differentiated in free osteogenic medium (O.D.). Ribosomal protein S14 gene was used as housekeeper. All data are means ± SD of three different experiments. The statistical analysis was determined by Student's t-test. ^∗^*p* < 0.05, ^∗∗^*p* < 0.01, ^∗∗∗^*p* < 0.001.

To test whether osteogenesis was activated by GM1 through the inhibition of PDGFR-*β*, hTSCs were induced to differentiate in the presence of the ganglioside and then subjected to PDGFR-*β* analysis by Western blot. Results revealed that GM1-treated cells showed a 40% decrease in PDGFR-*β* phosphorylation, measured as the pPDGFR/PDGFR ratio, as compared to untreated cells, supporting the hypothesis of a GM1-induced inhibition of PDGFR-*β* (Figure 3(a)). Furthermore, it was assessed whether exogenous GM1 was able to counteract PDGF-induced activation of PDGFR-*β*, which is known to inhibit osteogenesis [21]. To this purpose, hTSCs were induced to differentiate for 17 days in normal osteogenic medium in the presence of 10 ng/ml PDGF-BB, which caused a 43% decrease in ALP expression (Figure 3(b)) and a 40% decrease in osteocalcin expression by real-time PCR (Figure 3(c)). On the other hand, addition of 100 *μ*M GM1 to the osteogenic medium containing 10 ng/ml PDGF-BB completely restored the differentiation capability of hTSCs, as ALP and osteocalcin expression levels were comparable to differentiated untreated controls (Figure 3(b) and 3(c)).

## 4. Discussion

In this work, we investigated the role of gangliosides in the osteogenic differentiation of adult human tendon stem cells that we isolated and characterized for the first time from human supraspinatus tendons [4]. The method used for ganglioside pattern analysis required an initial metabolic radiolabeling of cell sphingolipids by adding [3-^3^H]-sphingosine in the culture medium that has been effectively used in our laboratories for many years [13–15]. As a result, cells synthesize radiolabeled sphingolipids that can be separated by HPTLC chromatography and accurately measured with a radiochromatoscanner. The use of metabolic radiolabeling significantly improves the sensitivity of the method, reducing the number of stem cells required for each analysis. Results demonstrated that the two main gangliosides of hTSCs, GM3 and GD3, increased and decreased, respectively, when cells were differentiated toward osteoblasts or adipocytes, suggesting that the modulation of these gangliosides is possibly linked to a general change of the biological status of the cell and not to the commitment toward a specific cell lineage. On the other hand, a marked increase of ganglioside GM1 was observed only during osteogenesis, supporting the possible role of this ganglioside in driving the process (Figure 1). The increase in GM1 content was accompanied by an increase of its synthase, which was instead reduced during adipogenesis (Figure 1). Interestingly, the addition of exogenous GM1 to the differentiation medium improved osteogenesis, as confirmed by a significant increase of ALP gene expression, which is a specific osteoblast marker, as well as by an increase of the extracellular matrix mineralization, as assessed by alizarin red staining (Figure 2). On the contrary, gene expression of the adipogenic markers PPAR-*γ* and LPL decreased upon GM1 supplementation to the adipogenic differentiation medium, supporting the idea that the ganglioside could inhibit the process (Figure 2). We then investigated the mechanism of GM1-induced increase of osteogenesis in hTSCs. Along this line, it has been reported that gangliosides can regulate the activity of the epidermal growth factor receptor [22], the fibroblast growth factor receptor [23], the nerve growth factor receptor (NGF) [24], the platelet-derived growth factor receptor (PDGFR) [25], and the insulin receptor (IR) [26]. In particular, it has been shown that GM1 is crucial in PDGFR regulation through different mechanisms of action that appear to be cell type-dependent. In this context, it has been demonstrated that, in fibroblasts, GM1 is able to inhibit the ligand-mediated phosphorylation of tyrosine residues of the cytoplasmic tail of the receptor [27], as well as the ligand-induced intracellular association of SH2-containing proteins with PDGFR in human glioma cells [28]. On the contrary, in Swiss-3T3 cells, it has been demonstrated that GM1-mediated inhibition of PDGFR requires the extracellular and/or the transmembrane domains of the receptor [29]. Moreover, in the same cell line, it has been shown that GM1 regulates PDGFR signaling by controlling the distribution of the receptor in- and outside of lipid rafts and that PAG regulates the membrane partitioning and the mitogenic signaling of PDGFR through an increase in GM1 levels in caveolae [30, 31]. PDGF/PDGFR signaling is reported to be involved in the regulation of various cell functions, including osteogenesis and adult stem cell differentiation toward osteoblasts. In particular, it has been observed that the downregulation of PDGR*α* promotes osteogenic differentiation of MSCs through the BMP/smad signaling pathway [32], and the blocking of the PDGFR-*β* pathway markedly promotes osteoblast differentiation and matrix mineralization in mouse osteoblastic MC3T3-E1 cells [33]. Moreover, PDGFR-*β* inhibition increases the osteogenic differentiation of primary rat osteoblastic cells [34] and human MSCs [21]. Altogether, these results support the hypothesis that GM1 could exert its effects on osteogenesis through the inhibition of the PDGF receptor also in hTSCs. To test this hypothesis, we assessed the activation levels of the PDGFR-*β* receptor during osteogenesis in the presence of exogenous GM1 in the culture medium. Indeed, we observed a significant decrease in the activation of the receptor when GM1 was added to the differentiation medium (Figure 3). To further confirm our hypothesis, we assessed whether GM1 was able to counteract the inhibition of osteogenesis caused by the activation of PDGFR-*β* upon addition of its ligand (PDGF-BB) in the differentiation medium. Results showed that PDGF-BB stimulation inhibited osteogenesis, as confirmed by a significant decrease of ALP and osteocalcin gene expression. As anticipated, the addition of GM1 to the osteogenic medium containing PDGF-BB completely restored the differentiation capabilities of hTSCs, as we could observe ALP and osteocalcin expression levels similar to untreated control cells (Figure 3).

## 5. Conclusions

In conclusion, our results show that ganglioside GM1 significantly increases during osteogenic differentiation of hTSCs. Most importantly, the ganglioside increase is instrumental for driving the process through the inhibition of PDGFR-*β*. Indeed, the addition of exogenous GM1 to the differentiation medium greatly increased the osteogenic capabilities of hTSCs, supporting its possible use as a new factor to be added in the differentiation medium to improve this process. Further studies are ongoing in our laboratories to fully elucidate the mechanism of GM1 regulation of PDGFR-*β* activation and the possible therapeutic application of GM1 in regenerative medicine.

## Data Availability

The data used to support the findings of this study are available from the corresponding author upon request.
